# Addressing bias and knowledge gaps regarding race and ethnicity in neonatology manuscript review

**DOI:** 10.1038/s41372-022-01420-7

**Published:** 2022-06-06

**Authors:** Kayla L. Karvonen, Elizabeth M. Bonachea, Heather H. Burris, Yarden S. Fraiman, Henry C. Lee, Alvaro Proaño, Valencia P. Walker, Margaret G. Parker

**Affiliations:** 1 Department of Pediatrics, University of California San Francisco, San Francisco, CA USA; 2 Neonatal Justice Collaborative (NJC), San Francisco, CA USA; 3 Division of Neonatology, Nationwide Children’s Hospital, Columbus, OH USA; 4 Division of Neonatology, The Children’s Hospital of Philadelphia, Philadelphia, PA USA; 5 Department of Neonatology, Beth Israel Deaconess Medical Center, Boston, MA USA; 6 Department of Pediatrics, Stanford University School of Medicine, Stanford, CA USA; 7 Department of Pediatrics, Tulane University, New Orleans, LA USA; 8 Department of Pediatrics, Ohio State University College of Medicine, Columbus, OH USA; 9 Boston Medical Center, Department of Pediatrics, Boston University School of Medicine, Boston, MA USA

**Keywords:** Ethics, Epidemiology

## Abstract

A recent shift in public attention to racism, racial disparities, and health equity have resulted in an abundance of calls for relevant papers and publications in academic journals. Peer-review for such articles may be susceptible to bias, as subject matter expertise in the evaluation of social constructs, like race, is variable. From the perspective of researchers focused on neonatal health equity, we share our positive and negative experiences in peer-review, provide relevant publicly available data regarding addressing bias in peer-review from 12 neonatology-focused journals, and give recommendations to address bias and knowledge gaps in the peer review process of health equity research.

## Introduction

In response to the murders of Breonna Taylor and George Floyd there has been a shift in public and academic attention to address racism, racial disparities, and health equity. Health equity is achieved when every person has the ability to attain their health potential. One of many major barriers to health equity include racism, or discrimination on the basis of one’s racial group. Racism can be individualized, internalized, and systemic and all forms contribute to racial disparities in health outcomes. Systemic racism is a form of racism that is embedded in laws, policies, and institutions, including academic medicine, that has resulted in a disparate distribution of goods, services, and opportunities for racial groups [1]. Despite an abundance of calls for papers addressing health equity in major journals, the extent that reviewers and editors are adequately trained to critically evaluate the use of social constructs, like race, in research studies is highly variable. A major contributor to this knowledge deficit is the historical false belief in race as a biological construct by the scientific community and a paucity of published articles naming racism as a major driver of racial disparities [2, 3]. Omission of rigorous research standards for evaluating race and racism has contributed to harmful rhetoric such as the biologic fallacy of race [4].

In addition to knowledge gaps by reviewers regarding the evaluation of social variables like race, explicit or implicit bias can occur in the manuscript review process [5, 6], which may be more epitomized during peer review of articles focused on health equity that use social variables in their approach. For example, microaggressions are a form of discrimination defined as “slights” that communicate a negative attitude toward marginalized groups. Microaggressions disproportionately impact marginalized groups and are commonplace in the workforce’s daily lives; peer-review is no exception [7]. In order to combat bias in reviews, scholars have suggested diversification of editorial boards, as well as intentional recruitment, education, and compensation of diverse pools of peer reviewers [4, 8, 9]. Others have called for explicit standards for evaluation of race and ethnicity [4, 10, 11]. In light of these concerns, more recently, some journals have established new author guidelines for addressing race and racism [5, 12–16]. However, standardized criteria have not been agreed upon or adopted for many academic journals.

As health equity researchers in academic neonatology, we offer [1]. Our own personal positive and negative experiences within the last two years that highlight knowledge gaps and bias in the peer-review process [2]; A brief summary of publicly available data from major, neonatal-focused journals regarding existing processes to evaluate health equity research and address bias in the review process; and [3] Our recommendations for neonatology-focused journals regarding these aforementioned issues.

## Our experiences in peer-review of neonatology-focused journals

### Knowledge gaps during peer-review

“After submitting a study for peer review that tracked hospital practices by race/ethnicity and language, a reviewer argued there was no rationale as to why such disparities in hospital practices could exist and questioned why we chose to examine this. Denial of disparities in care quality by this reviewer suggested a significant knowledge gap of long-standing literature. I alerted my concern to the editor who omitted this review and sent it out to a different reviewer.”

“When exploring the experience of traditionally marginalized communities in a qualitative study, a reviewer suggested that to increase the validity of the study, we should compare the experience to the majority’s experience. Centering whiteness and white normativity was problematic in a study designed to center at the margins [17]. Following this comment, our team opted to include prose in the discussion about findings of previous studies focused on white populations. It was eventually published.”

“As a peer reviewer I suggested capitalizing the ‘b’ in Black when identifying race and to not use ‘Blacks’ when referring to Black persons. The authors responded that they preferred not to edit for readability, despite the possibility of offensive interpretation and accepted terminology.^12^ Upon re-review, the editor agreed and sent me a positive reply acknowledging the ‘teaching moment’ for the authors.”

### Bias in the peer-review process

“I revealed my identity in a commentary and received an inappropriate comment during the review process. I did not know where to anonymously report my experience of discrimination to avoid worrying about my future relationship with the journal.”

## Evaluation

To better understand the extent that journals serving the academic neonatology audience have guidance regarding evaluation of social variables, like race and ethnicity, in articles and processes to address bias in peer-review, we examined the websites of 12 major academic journals that publish in neonatology. Journals were chosen by combining our searches of academic journals with high frequency of neonatal-perinatal material based on a PubMed query (currently utilized by neopapers, an automated literature Twitter account that has been created to publish recent articles with content related to neonatology and an active account in the #neoTwitter community [18, 19]), and authors’ familiarity. Journal characteristics were created by the authors to evaluate previous commitment to health equity topics, transparency of diversity, equity, and inclusion issues, intention to diversify editorial staff, and existence of an anonymous system of reporting discrimination in peer review. No formal recommendations or regimented criteria exist to evaluate journal processes for inclusion of health equity content or bias in review, thus our evaluation metrics were developed through iterative discussion by authors and guidance from previous literature [2–4, 8–10].

We summarize findings in Table 1. We found that more than 75% of journals have published at least one original research, commentary, and perspective piece on health equity since journal conception, suggesting recognition of addressing social variables in neonatology journals. Regarding processes which may improve bias in peer-review more broadly, no journal had readily available data on racial, ethnic, or gender diversity of reviewers, editors, editorial board, but four (33%) had a statement of current efforts to diversify reviewers, editors, and/or editorial boards and only one (8%) journal provided information for how to apply to be an editorial board member on their website. Only one (8%) journal had a statement separate from Committee of Publication Ethics (COPE) guidelines for how to address bias in peer-review. COPE is an organization dedicated to providing resources and leadership on publication ethics which has recently published guidance on addressing bias in peer review. Although contact information was nearly always available for both editor-in-chief and members of the editorial board as a potential pathway to report discrimination (83% and 100% respectively), we could find no evidence of journals with a transparent system of anonymous peer review feedback to report racism, bias, or discrimination on their website.

**Table 1:** Summary of processes to address evaluation of health equity-oriented articles and bias in peer review among neonatal-focused academic journals^a^.

Journal processes	Journal number												Total *N* (%)
	1	2	3	4	5	6	7	8	9	10	11	12	
Evidence of manuscripts focused on health equity per current website^b^													
Research paper published on health equity	Yes	Yes	Yes	Yes	No	No	^c^	Yes	Yes	Yes	Yes	Yes	9 (75%)
Call for manuscripts or special issues focused on racial disparities or structural racism	Yes	No	No	No	No	No	No	No	Yes	No	Yes	Yes	4 (33.3%)
Invited commentaries for manuscripts focused on health equity	Yes	Yes	No	Yes	Yes	Yes	Yes	Yes	Yes	Yes	Yes	Yes	10 (83.3%)
Perspective or editorial published on health equity	Yes	Yes	No	Yes	No	No	Yes	Yes	Yes	Yes	Yes	Yes	9 (75%)
Evidence of processes to address bias in peer review per current website^b^													
Statement on website on how journal may approach bias in peer-review	No	No	No	No	No	No	No	No	No	No	No	Yes	1 (8.3%)
Transparent process by which authors may respond to concerns in their review	No	No	No	No	No	Yes	Yes	Yes	No	No	Yes	Yes	5 (42.7%)
System to report perceived racism and/or other kinds of discrimination during the peer-review process anonymously	No	No	No	No	No	No	No	No	No	No	No	No	0 (0%)
Transparent application process for selection on editorial board	No	No	No	No	No	No	No	No	No	No	No	Yes	1 (8.3%)
Contact information for editor-in-chief readily available	Yes	Yes	Yes	No	Yes	Yes	Yes	Yes	Yes	Yes	No	Yes	10 (83.3%)
Contact information for editorial board (not editor-in-chief) readily available	Yes	Yes	Yes	Yes	Yes	Yes	Yes	Yes	Yes	Yes	Yes	Yes	12 (100%)
Transparency of race/ethnicity, gender or other characteristics of reviewers, editors-in-chief, or other editorial staff	No	No	No	No	No	No	No	No	No	No	No	No	0 (0%)
Statement on effort to diversify reviewers, editors-in-chief, or other editorial staff	No	No	No	Yes	Yes	Yes	No	No	No	No	No	Yes	4 (33.3%)

^a^Reference to COPE website and guidelines was not sufficient to account for any criteria above.

^b^Data acquired from corresponding websites January 5–13, 2022.

^c^n/a, not within journal scope.

## Discussion

Our anecdotal experience and review of publicly available data from journal websites suggest that there is room for improvement to address knowledge gaps in peer-review of neonatology articles focused on health equity, which often utilize social variables like race and ethnicity in their methodology and therefore may increase potential for bias in the peer-review process. With heightened national attention on the role of race, racism, and other social factors on health outcomes, we anticipate that research in this area will continue to grow. Therefore, journal guidelines for authors and reviewers are needed to educate the neonatal research community and set standards on use of race and racism in research. While our experiences focus primarily on the social construct of race and ethnicity, we believe that our experiences and our recommendations may impact those doing research in other domains that also utilize social variables such as income, primary language, and immigration status.

Researchers also must be protected from discrimination and bias in the peer-review process. Few journals have made transparent efforts to diversify staff or develop mechanisms for providing anonymous feedback in the setting of perceived racism and discrimination in the review process. In our review, many journals have statements demonstrating commitment to adhere to COPE guidelines, which recently organized a Diversity Equity and Inclusion (DEI) committee that has provided resources and a commitment to addressing ethics and DEI for journals [20]. A few journals we evaluated have also signed the joint commitment for action on inclusion and diversity in publishing, launched in June 2020 by the Royal Society of Chemistry with ongoing efforts to set minimum standards for inclusion and diversity for scholarly publishing. Planned efforts include, but are not limited to, setting minimum targets to achieve diverse representation of authors, reviewers, and editorial boards, developing language standards, reviewing and revising editor and editorial board member selection processes, and publicly reporting their progress [21]. We are encouraged by the intention and progress made by several journals and publishing bodies, and hope to see fully transparent standards for DEI in the peer-review process across all neonatology publishing journals.

We consider the diversification of reviewer, editorial boards, and editors to be of particular importance for the health equity publication process in neonatology journals. Not only does the inclusion of perspectives of lived experience and participation in scholarly health equity activities advance the quality of work in our field, it also begins to address historical exclusion of minoritized individuals from scientific discourse [8, 10]. In our field, there continues to be underrepresentation of minoritized trainees and physicians scientists [22]. Harm during the peer review process can negatively impact the pursuit of antiracism and health equity work and disproportionately impacts minoritized researchers [10, 23]. Building infrastructure for transparency and accountability is necessary for ongoing publication of high quality health equity research [10]. We hope that our recommendations on how to improve the peer-review process in neonatology journals can help improve the trust of neonatal researchers and mitigate systemic inequities in publication in research focused on health equity.

Our review was limited to information readily available on journal websites. This may not fully encompass efforts made by academic journals to support health equity research and address bias in the peer-review process. The journal processes evaluated were designed by authors and thus are not previously validated and may not sufficiently evaluate the performance of journals. Our perspective piece does not compare the performance of neonatology journals, which tend to be clinically focused, to social science or public health focused journals. Performance in neonatology journals may be different from journals dedicated specifically to health equity. Regardless we see importance in addressing bias and knowledge gaps within our field while understanding challenges may be different or similar to other fields.

## Recommendations

We offer the following recommendations to improve the peer-review process:

- A standard of proficiency of reviewers in evaluation of social variables and constructs, including race and racism. While there are a few resources available that address this topic [4, 12–15, 24], it is unclear what standard exists or should be followed among neonatology journals. At minimum, we recommend statements that confer that race is a social construct without biological basis and explicitly stating racism as a primary etiology of racial disparities.
- Transparency of current demographics of authors, reviewers, editorial boards, and editors. Although lack of diversity in academia is a widely known problem and we suspect it is no different in neonatology, the demographics of the participants in the peer-review process was not explicitly stated in the journals reviewed. Transparency offers a route towards accountability.
- Diversification of reviewers, editorial boards, and editors with transparent, publicly announced target dates and goals.
- Transparency, evaluation, and equal opportunity of editorial board selection process to facilitate diversification.
- Recruitment and appropriate compensation for subject matter experts for time. We are not aware of any current resources or guidelines that define subject matter expertise prior to review. In the case of health equity research, lived experience should be recognized as a form of subject matter expertise. Similarly, we are not aware of resources to guide overall reviewer recruitment nor compensation for reviewers by journals. If compensation for peer review is not provided by journals, institutions should consider ways in which to support faculty and trainees who participate in the peer-review process through financial incentives, promotion, or other forms of meaningful recognition.
- Standardized and robust training on an antiracism and the measurement and evaluation of social constructs in academic medicine and biomedical research that begins early and continues throughout professional careers.
- Anonymous reporting mechanism for authors to report racism and/or other types of bias in the peer-review process

## Data Availability

All data generated and analyzed during this study are included in this published article.
